# Structural barriers to medically indicated abortion in Germany: A qualitative study of provider perspectives

**DOI:** 10.1186/s12978-025-02116-9

**Published:** 2025-12-10

**Authors:** Amelie Kolandt, Susanne Michl, Mirjam Faissner

**Affiliations:** Institute of the History of Medicine and Ethics in Medicine, Charité - University Medicine Berlin, Thielallee 71, Berlin, 14195 Germany

**Keywords:** Medically indicated abortion, Abortion access, Abortion law, Healthcare barriers, Abortion stigma, Reproductive rights, Patient-centred care, Germany

## Abstract

**Background:**

In Germany, to perform an abortion after 14 weeks of gestation, physicians are legally required to provide a medical indication due to a serious risk to the pregnant person’s life or health. This study explores legal, institutional, and societal factors influencing abortion care in Germany from the perspective of abortion providers and counsellors.

**Methods:**

We conducted 42 semi-structured expert interviews with gynaecologists and abortion counsellors across Germany. Data were analysed using Mayring’s qualitative content analysis with a mixed inductive–deductive approach.

**Results:**

In our analysis, we identified four overarching themes that influence care for medically indicated abortions in Germany:

(1) Varying Availability and the Right to Refuse: Abortion services in Germany are unevenly distributed, with notable urban–rural divides. This restricts procedural options and leads to delays. The experts raised concerns over institutional refusals by entire hospital departments or publicly funded facilities.

(2) Legal Regulation and Stigmatisation: Although medically indicated abortions are legal, their regulation under the Criminal Code fosters a sense of criminality among providers. Stigma can negatively affect professional support for patients in their decision either to terminate or continue a pregnancy.

(3) Lack of Training and Standardisation: Abortion care is underrepresented in medical education and obstetric training, with few opportunities to acquire the necessary skills. The lack of national clinical guidelines further contributes to inconsistent interpretations of the medical indication and intransparency of processes.

(4) Intersectional Aspects: Systemic inequities disproportionately affect structurally marginalised groups—including those living in rural areas, individuals with migration histories, language barriers, mental health conditions, or prior trauma. Experts described how opaque medical indication criteria, stigma, and lack of trauma-sensitive care result in delayed, inaccessible, or denied care.

**Conclusion:**

In Germany, abortions on medical grounds are complicated by legal ambiguities, fragmented care provision, and frequent use of the right to refuse. While the right to refuse protects providers, its opaque application can exacerbate inequalities and delay access to care. Stigma – legal, institutional, and interpersonal – remains a major barrier, reinforced by the Criminal Code, inadequate training, and generational differences among healthcare professionals. Future legal frameworks should guarantee equal access to care irrespective of social and medical factors. National clinical guidelines on abortion beyond 14 weeks’ gestation and bereavement care are urgently required.

**Supplementary Information:**

The online version contains supplementary material available at 10.1186/s12978-025-02116-9.

## Abstract (de)

**Hintergrund:**

In Deutschland sind Ärzt*innen verpflichtet, für einen Schwangerschaftsabbruch nach der 14. Schwangerschaftswoche eine medizinische Indikation aufgrund einer ernsthaften Gefahr für das Leben oder die Gesundheit der schwangere Person anzugeben. Diese Studie untersucht rechtliche, institutionelle und gesellschaftliche Faktoren, die die Versorgung mit Schwangerschaftsabbrüchen in Deutschland aus Perspektive von Ärzt*innen und Schwangerschaftskonfliktberater*innen beeinflussen.

**Methoden:**

Wir führten 42 halbstrukturiere Expert*inneninterviews mit Gynäkolog*innen und Schwangerschaftskonfliktberater*innen in ganz Deutschland durch. Die Daten wurden mit Hilfe einer qualitativen Inhaltsanalyse nach Mayring im gemischt induktiv-deduktiven Ansatz ausgewertet.

**Ergebnisse:**

In unserer Analyse haben wir vier übergeordnete Themen identifiziert, die die Versorgung bei medizinisch indizierten Schwangerschaftsabbrüchen in Deutschland beeinflussen:

(1) Unterschiedliche Verfügbarkeit und das Recht auf Verweigerung: Schwangerschaftsabbrüche sind in Deutschland ungleich verfügbar, wobei es erhebliche Unterschiede zwischen Stadt und Land gibt. Dies schränkt die Wahlmöglichkeit bezüglich der Methode ein und führt zu Verzögerungen. Die Expert*innen äußerten Bedenken hinsichtlich institutioneller Verweigerungen durch ganze Krankenhausabteilungen oder öffentlich finanzierte Einrichtungen.

(2) Rechtliche Regelung und Stigmatisierung: Obwohl medizinisch indizierte Schwangerschaftsabbrüche legal sind, fördert ihre Regelung im Strafgesetzbuch ein Gefühl der Kriminalität bei den Anbieter*innen. Stigmatisierung kann sich negativ auf die professionelle Unterstützung von Patient*innen bei ihrer Entscheidung für oder gegen einen Schwangerschaftsabbruch auswirken.

(3) Unzureichende Ausbildung und Standardisierung: Schwangerschaftsabbrüche sind in der medizinischen Ausbildung und in der geburtshilflichen Weiterbildung unterrepräsentiert und es gibt nur wenige Möglichkeiten, die erforderlichen Fähigkeiten zu erwerben. Das Fehlen nationaler klinischer Leitlinien führt zusätzlich zu uneinheitlichen Interpretationen der medizinischen Indikation und intransparenten Prozessen.

(4) Intersektionale Aspekte: Systemische Ungleichheiten betreffen strukturell marginalisierte Gruppen in unverhältnismäßigem Umfang – darunter Menschen in ländlichen Gebieten, Menschen mit Migrationgeschichte, Sprachbarrieren, psychischen Erkrankungen oder Traumata. Expert*innen beschreiben, wie undurchsichtige medizinische Indikationskriterien, Stigmatisierung und mangelnde traumasensible Versorgung zu verzögerter, unzugänglicher oder zur Verweigerung von Versorgung führen.

**Fazit:**

In Deutschland werden Schwangerschaftsabbrüche aus medizinischen Gründen durch rechtliche Unklarheiten, eine fragmentierte Versorgung und die häufige Inanspruchnahme des Verweigerungsrechts erschwert. Das Verweigerungsrecht schützt zwar die Ärzt*innen, seine undurchsichtige Anwendung kann jedoch Ungleichheiten verschärfen und den Zugang zur Versorgung verzögern. Stigmatisierung – rechtlich, institutionell und interpersonell – bleibt ein großes Hindernis, das durch das Strafgesetzbuch, unzureichende Weiterbildung und Generationsunterschiede innerhalb des Fachpersonals noch verstärkt wird. Künftige rechtliche Rahmenbedingungen sollten einen gelichberechtigten Zugang zur Versorgung unabhängig von sozialen und medizinischen Faktoren gewährleisten. Nationale klinische Leitlinien für Schwangerschaftsabbrüche nach der 14. Schwangerschaftswoche und für die Trauerbegleitung sind dringend erforderlich.

## Plain English Summary

This study looks at the challenges people in Germany face when trying to access an abortion after 14 weeks of pregnancy, which is only allowed if there’s a serious risk to the person’s life or health. Researchers interviewed physicians and counsellors across the country to understand what makes it difficult to get this type of care. They found four main problems:

(1) Limited availability: Abortion services are not available everywhere, especially in rural areas. Many physicians are retiring, and fewer are taking their place. Some hospitals, including publicly funded ones, refuse to provide abortions altogether, causing delays and reducing options for patients.

(2) Legal and social pressure: Although these abortions are legal, they are still treated under criminal law, which makes physicians feel uncertain and can lead to feelings of shame. Some patients also face judgement based on ideas about “health” or disability.

(3) Lack of training: Abortion is rarely covered in medical training, so many physicians don’t learn the necessary skills. There are also no clear national guidelines, which means practices vary a lot across the country.

(4) Inequality: People from marginalised backgrounds struggle the most, such as those living in remote areas or with language or mental health challenges. The regulations and lack of support can make it even harder for them to access care.

The study calls for clearer laws, better training, and national standards to make abortion care more equal and accessible for everyone.

## Introduction

A ‘medically indicated abortion’ refers to the termination of a pregnancy based on medical reasons related to the pregnant person’s health. Depending on accessibility of abortion services and experiences of stigmatisation, the process can lead to emotional and mental distress for patients [1, 2]. Access barriers are particularly detrimental to patients who face multiple forms of marginalisation, such as racism, poverty, or health-related issues [3–6] or due to gender identity [7–9]. Identifying current challenges in the provision of medically indicated abortions in Germany is of high relevance to providing patient-centred care.

### Legal regulation of abortion in Germany

In Germany, abortion is prohibited under § 218 of the Criminal Code [10]. 95% of abortions occur under the ‘counselling regulation’ (German: ‘Beratungsregelung’), which means that the procedure is exempt from punishment after mandatory counselling and within a limit of 12 weeks post-conception (p.c.). The German Criminal Code allows exceptions for medical and criminological indications [10]. The criminological indication allows abortion up to 12 weeks p.c. in cases of sexual violence.

Until 1995, abortion was legal under the ‘embryopathic indication’, which referred to cases in which a continuation of the pregnancy could not be expected based on the “child’s irremediable impairment of its state of health” [11]. The Federal Constitutional Court ruled the embryopathic indication to be unconstitutional because it conveyed negative judgements about life with a disability and supported ableism, i.e. discrimination against disabled people [12].

Since the abolition of the embryopathic indication in 1995, a termination of pregnancy beyond the 14th gestational week is only possible on the basis of a medical indication. A medical indication can be issued if ‘considering the pregnant woman’s present and future circumstances, the termination is medically necessary to avert a danger to the life of or the danger of grave impairment to the pregnant woman’s physical or mental health and if the danger cannot be averted in another manner which is reasonable for her to accept’ [10]. These conditions can be fulfilled in case of a foetal illness or potential disability [13, 14].

Two independent physicians must be involved: one issuing the indication, another performing the procedure. The involvement of ethics committees is possible, but not legally required, nor is mandatory counselling [15]. There are no gestational age limits for medically indicated abortions [16, 17].

In 2024, a government-appointed expert commission recommended regulating abortion outside of criminal law to improve access to care and support reproductive autonomy [18]. It advises the legislator to revise the medical indication, as the current practice lacks transparency: According to the commission, there is no statutory criteria defining under which conditions an abortion is permissible in the case of a prenatal diagnosis, nor which alternative options are considered reasonable to avert danger to the pregnant person’s life or health [18].

Until its repeal in 2022 following widespread criticism, § 219a of the Criminal Code prohibited physicians from publicly stating (e.g. on their website) that they provide abortion services [19]. Despite the legal change, current research shows that the digital information landscape still lacks information provided by healthcare professionals themselves [20]. § 219b of the Criminal Code forbids the distribution of items “intended for use in abortions” [10], including abortion medication, thereby subjecting medication abortion to additional access restrictions.

No one may be compelled to take part in an abortion. This includes its performance, anaesthesia, and nursing care. We use the term ‘*right to refuse’* in our study, because German legislation does not explicitly require the refusal to be based on conscientious grounds [21]. Participation in abortion can be declined without justification, unless the life or health of the pregnant person is in immediate danger. This sets it apart from the international discourse, where the term “conscientious objection” is commonly used, typically referring to ethical or moral grounds [22].

### Prenatal diagnostics in Germany

Prenatal diagnostics are an optional part of antenatal care in Germany aimed at identifying “genetic conditions, congenital anomalies, and developmental disorders” as explained in the patient information published by the Federal Institute for Public Health in 2024 [15]. The regulatory framework aims to ensure informed consent through mandatory counselling and medical explanation prior to any procedure. Medical professionals must inform patients about the nature, risks, and potential consequence of each test. These include standard ultrasound scans each trimester, first-trimester screening, and – if medically indicated – non-invasive prenatal testing (NIPT) for trisomy 21, 13, and 18 as well as chorionic villus sampling, amniocentesis, and cordocentesis. The routine ultrasound examinations are covered by health insurance, additional diagnostic procedures, if they are indicated [15]. According to German law, families are legally entitled to free, confidential, and anonymous counselling [23]. This includes information on available legal benefits and support services, strategies for addressing psychosocial challenges related to pregnancy, and support options for disabled people and their families. The right to counselling also includes follow-up support after an abortion or childbirth. In case the patient choses to terminate a pregnancy, the prenatal diagnostician providing the diagnosis is legally not allowed to perform the abortion [23].

### Prevalence and abortion care landscape in Germany

Between 2013 and 2022, medically indicated abortions accounted for an annual average of 3.8% of all abortions in Germany: 24.8% of them take place in the first trimester, 58.2% in the second and 16.9% in the third trimester [24]. Approximately 80% of *all* abortions in the first trimester (irrespective of indication) are performed in the outpatient sector and approximately 20% in the inpatient sector [24–26]. Second and third trimester abortions are commonly performed in the inpatient sector [27, 28]. As most terminations on medical grounds occur later in pregnancy, this study mainly focuses on second– and third-trimester abortions.

The *United Nations* (UN) and *World Health Organisation* (WHO) advice for a de-criminalisation of abortion without grounds-based regulations [29]. They define key criteria for health care services such as abortion: (1) availability, (2) accessibility, (3) acceptability, (4) quality, and (5) equity, inclusivity, and patient-centredness of abortion care [29]. In the following, we summarise international data on abortion care in relation to these criteria, and contextualise existing data on the German landscape accordingly.

#### Availability and accessibility

To follow UN standards, states must ensure sufficient *availability* of healthcare facilities, services, and essential medicines, including (emergency) contraceptives and abortion medication, and ensure their physical and economical *accessibility* without discrimination [29, 30]. International reviews highlight common barriers to abortion care: criminalisation imposes legal and financial obstacles, a shortage of trained providers limits access, and negative attitudes among healthcare professionals restrict care [31, 32]. For instance, de Londras et al. argue that “criminalisation results in a ‘chilling effect’ in the provision of healthcare, with negative implications for the rights to life, health and privacy of women who seek abortion care” [33]. Also, conscientious objection can negatively impact availability of abortion care, and the UN advises against its institutional invocation [22].

In Germany, different deficits of abortion care have been identified: Between 2003 and 2024, the number of institutions reporting to perform abortions in Germany fell by nearly 47% from approximately 2050 institutions to 1100, indicating a reduction in availability of the service [34]^1^. A 2021 mixed-methods study on second– and third-trimester abortion care demonstrated various access barriers, including poor coordination between counselling centres, outpatient physicians, and abortion facilities [35]. The study also reported that some facilities defined the involvement of ethics committees as a mandatory institutional standard despite it not being mandatory by law [35]. Overall, legal barriers and provider shortages push some German patients to seek abortions abroad [35]. In 2017, German residents comprised the largest group of foreign patients receiving abortion care in the Netherlands [36].

#### Quality of care

To meet UN standards of *quality*, reproductive healthcare services must be scientifically and medically appropriate, requiring professionally trained staff, evidence-based protocols, and approved drugs and instruments [30]. Medical standards for abortion vary by gestational age. While Germany lacks official guidelines for medical indications or abortions beyond the first trimester, the WHO recommends medication abortion or dilatation and evacuation (D&E) beyond 14 weeks’ gestation [29]. D&E is a method that is safe and effective for induced abortions and septic abortions [37], therefore reducing pregnancy-associated risk of mortality [38]. The *Royal College of Obstetricians & Gynaecologists* advises performing a foeticide by intracardial potassium chloride injection from 22 gestational weeks onwards [39]. An alternative in cases of a non-viable foetus is palliative birth, where preterm labour is induced, followed by neonatal palliative care [40].

Data from the Federal Statistical Office points to shortcomings in the quality of abortion care in Germany. For example, Dilatation and Curettage (D&C) is still commonly used during first-trimester abortions [28], despite international recommendations favouring vacuum aspiration. Most abortions beyond 14 weeks’ gestation are performed medically; however, no detailed information is available on the procedures used in surgical abortions or in foeticides.

#### Acceptability of care

Respectful, patient-centred care should consider cultural, gender, age, and minority factors in order to increase the *acceptability* of care [29]. The WHO emphasises dignity, autonomy, equality, confidentiality, communication, social support, and trust as the foundation of inclusive, patient-centred abortion care. Holten et al. identified abortion stigma as an “institutionalisation of taboo in abortion law and care“ and a key barrier to care [41]. An international systematic review confirms that both patients and providers face stigma [42]. Millar points out that abortion stigma should be understood as a social process and “as a classificatory form of power that works through designating relations of difference” [43]. For a German context, studies raise worries about the acceptability of abortion care, especially beyond 14 weeks of gestation: For instance, people were found to experience high levels of stigma if their foetus received a prenatal diagnosis [44]. Other studies suggest patient-centredness of German abortion services during the first trimester is limited due to stigma, provider shortages, and inadequate information [45], and that barriers are compounded for rural residents, patients with migration history, and socioeconomically disadvantaged groups [46].

### Aim of the study

No study to date has examined the structural conditions of abortion care in Germany for medically indicated abortions in light of UN standards [47]. This qualitative study draws on the perspectives of professionals from a range of practical and regional backgrounds to examine the following research questions: How do gynaecologists and abortion counsellors in Germany experience the provision of medically indicated abortion care? What challenges do they see? To what extent do existing care structures meet international standards of patient-centred reproductive healthcare?

## Material and methodology

### Study design and framework

This qualitative study involved 42 expert telephone interviews conducted between September 2020 and May 2021 at *Charité - University Medicine Berlin*. It was approved by the Ethics Research Committee of *Charité - University Medicine Berlin* (Registration Number EA2/013/20). The description of the methods and results follow COREQ criteria [48]. For the first leg of the study, we focused on the perspectives of gynaecological and counselling experts, whose central roles within the healthcare system provide valuable insights into the structures of abortion care in Germany. A second phase of the study, examining patients’ experiences with abortion provision in Germany, is currently underway.

We used Mayring’s method of content analysis [49] which allowed a detailed examination of current practices, challenges, and the interrelation of social, legal, and medical aspects in abortion care. The analytical framework for this study draws on healthcare evaluation criteria proposed by the WHO and the UN as cited above [29].

In addition, this framework is extended by incorporating the concept of structural stigmatisation. As outlined by Link and Phelan [50, 51], we understand abortion stigma as a social process that involves labelling, stereotyping, separation, status loss, and discrimination. It can restrict access to essential resources and services, and may be internalised, leading to feelings of shame, self-stigma, and the avoidance of healthcare. For a more detailed description of the methodology also compare Appendix 1.

### Recruitment

Experts were recruited across Germany via written enquiries to: (1) professional associations for gynaecologists and general practitioners, (2) abortion counselling providers, (3) state health authorities, (4) university hospital gynaecology and obstetrics departments, and (5) gynaecological health organisations through print/online advertisements and snowball sampling. All counsellors and physicians with professional expertise in abortion were invited to participate, regardless of their personal stance and whether or not they performed abortions themselves. Interested experts contacted AK via email and received verbal and written study details, including procedural and data security aspects. Participants provided written consent and could withdraw at any time.

### Sample description

We included 42 experts, comprising 20 abortion counsellors and 22 physicians. Most interviewed physicians (*n* = 20) had completed specialist training in gynaecology and obstetrics; *n* = 2 were in the final third of their residency in gynaecology and obstetrics. The majority of physicians interviewed were working in the outpatient sector at the time of the interview (*n* = 12). Three of them were providing abortion counselling, four medication abortion and five medication and surgical abortion during the first trimester respectively. Nine experts were working in the inpatient sector at the time of the interview. Three of them were providing medication abortion at all gestational limits and surgical abortion during the first trimester, two of them were providing medication and surgical abortion in the first and second trimester and four were providing abortion irrespective of method and gestational age. One physician in residency was seeking a new workplace to get abortion training at the time of the interview.

Four of the interviewees working in gynaecology had less than 10 years working experience. The majority of the gynaecological experts had 10 years or more of professional experience: Respectively nine participants had been working between 10 and 20 years or 20 years and more in gynaecology.

**Fig. 1:**
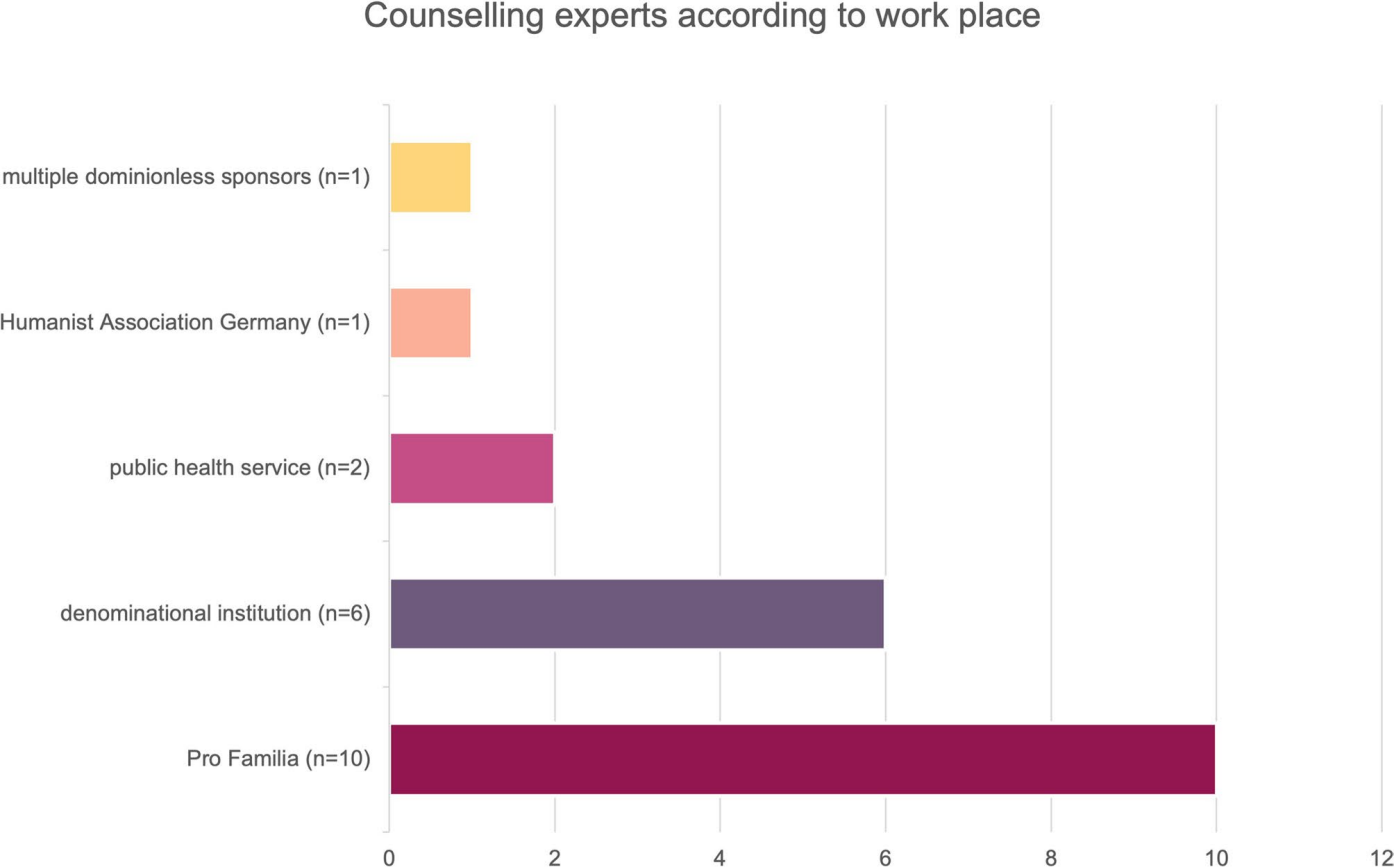
Counsellors according to their affiliations: The majority of counsellors interviewed (*n*=10, 50%, berry) were working at Pro Familia at the time of the interview. Counsellors working at a denominational institution made up the second largest group (*n*=6, 30%, purple). Two counsellors were working at public health service facilities (10%, pink) and respectively one counsellor was working for the Humanist Association Germany (5%, apricot) and a facility with multiple non-denominational sponsors (5%, yellow)

**Fig. 2:**
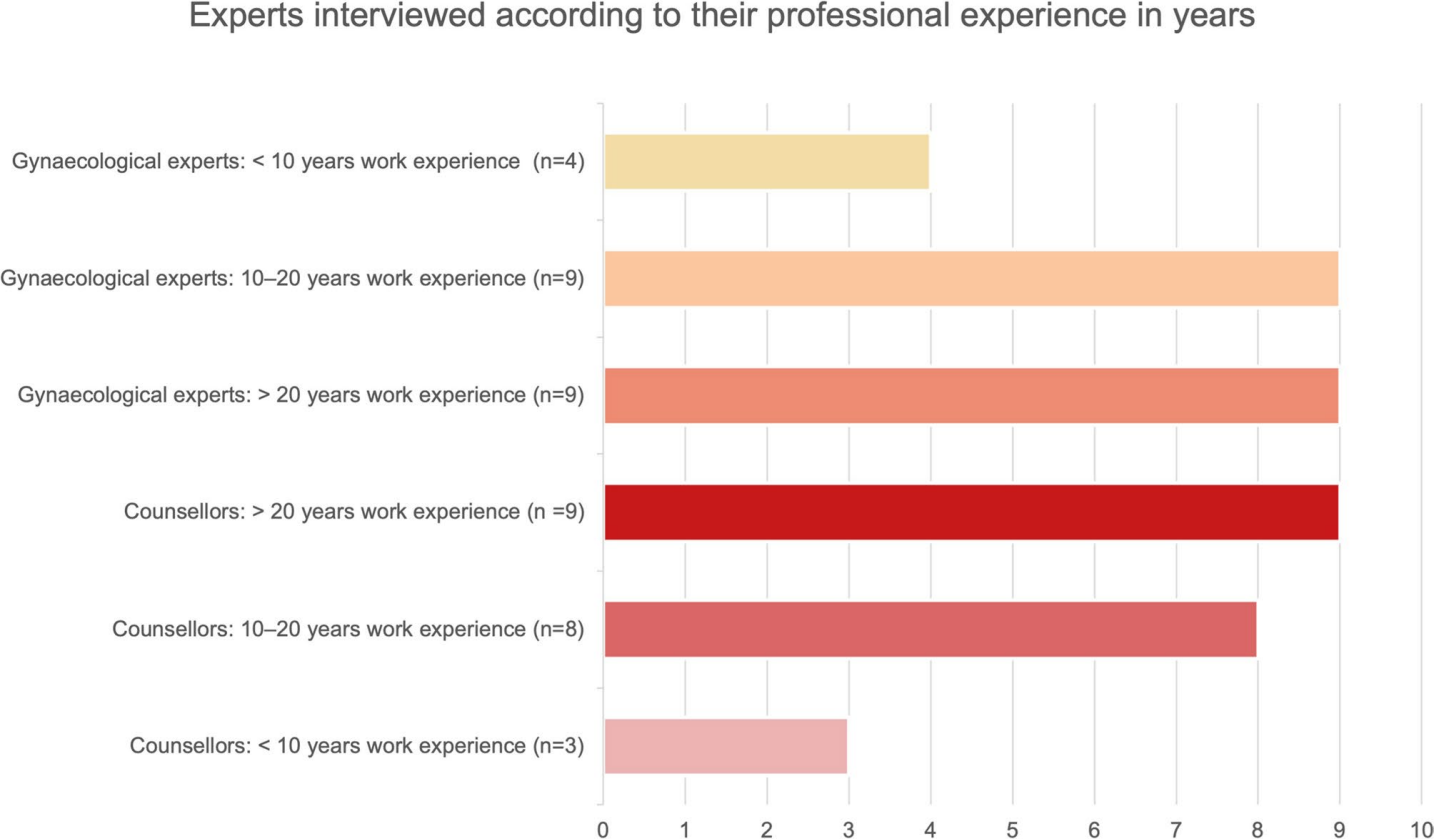
Experts according to their professional experience in years: Four of the interviewees working in gynaecology had less than 10 years working experience (10%, light yellow). The majority of the gynaecological experts had 10 years or more of professional experience: Respectively nine participants had been working between 10 and 20 years (22%, light orange) or 20 years and more (dark orange, 21%) in gynaecology. Most of the counsellors had 10 or more years of working experience at the time of the survey. Nine of them (21% of all experts, dark red) had 20 years or more experience in working as a counsellor, eight of them (19%, red) between 10 and 20 years. Three counsellors counted less than 10 years in working experience (7% of all experts, rosé)

Counsellors had diverse professional backgrounds (*n* = 9 social pedagogy, *n* = 5 social work, *n* = 4 psychology, *n* = 2 medicine—the latter working primarily as counsellors) and worked at non-denominational (*n* = 14) or denominational (*n* = 6, see Fig. 1) workplaces. The experts varied in geographical access to care (urban vs. rural), work experience (see Fig. 2) and were based in different federal states.

### Data collection

We conducted semi-structured interviews following Helfferich’s approach [52]. Interview guides covered the following topics: (1) motivation and role in abortion care, (2) daily work, challenges, solutions, (3) educational background, (4) state of care in Germany, and (5) future needs and visions (see Appendix 2). The interview guides were designed to cover the topic of abortion care in Germany in a broader sense, without limitations to indications or reasons for abortion. They were developed by AK and refined based on four pilot interviews. Additionally, participants completed a socio-demographic questionnaire on age, gender, education, training, and professional experience (see Appendix 2). The interviews were conducted by AK in German and lasted between 25 and 105 min (average: 50 min). They were audio-recorded, transcribed verbatim using an AI-based transcription service (*Happyscribe)*, manually corrected, and pseudonymised. All interviewees were offered to check the transcripts but none of them made use of the offer.

### Data analysis

We used the software *MaxQDA 2022* (VERBI Software GmbH, Berlin, Germany) to perform a mixed inductive-deductive content analysis: This comprised the steps of (1) paraphrasing, (2) abstraction, (3) inductive code derivation from the material, and (4) sub-category formation. For the structuring of the category system (step 4), we assigned developed sub-categories to the already existing WHO categories availability, accessibility, acceptability, quality, equity, inclusivity and patient-centredness in an deductive approach. The approach of combining the pre-existing WHO categories with inductive categories based on our interview material allowed to apply and refine the WHO framework. We achieved thematic saturation when no further sub-categories emerged, indicating that additional data were unlikely to generate new insights.

For intersubjective validation, two qualitative researchers at *Charité – University Medicine Berlin* cross-coded approximately 25% of the data using AK’s category system. Discrepancies were discussed until consensus was reached. Ambiguous interpretations were discussed in qualitative research colloquia at *Charité – University Medicine Berlin* and *Medizinische Hochschule Berlin Brandenburg*. The category system was revised with SM and MF and applied to the full dataset. Quotes were translated via the AI based-translation software *DeepL* and verified with forward–backward translation. The large language model *ChatGPT* was used for language refinement of the manuscript.

### Study team and reflexivity

The interviews were conducted by AK, who had prior qualitative research experience. Before data collection, she completed basic and advanced qualitative research courses at *Charité - University Medicine Berlin*. From 2017 until 2021, she was a volunteer member of *Medical Students for Choice Berlin*. Scientific supervision was provided by SM and MF, with expertise in literary studies, medical humanities, medicine, and philosophy. Both have extensive qualitative research experience. We acknowledge access to safe and legal abortion as a human right as defined by the United Nations, and highlight its significance for reproductive health and gender equality. To critically address this personal stance, we regularly discussed it during the research process in qualitative research colloquia.

## Results

We identified 4 main themes influencing the availability, accessibility, acceptability, quality as well as equity, inclusivity and patient-centredness of medically indicated abortions in Germany: (1) varying availability and the right to refuse, (2) the legal framework and stigmatisation, (3) the lack of training and standardisation, and (4) intersectional aspects. The following sections are structured around these main themes. To highlight how these themes influence the WHO criteria of abortion care in Germany, Tables 1, 2, 3 and 4 provide exemplary quotes (Eq). The asterisks (*) indicate sub-/categories that have already been described by the WHO. Additional sub-/categories developed by the authors of this study are marked with a plus (**+**).

**Table 1:** Main theme ‘varying availability and right to refuse’

**Categories and s ub-categories**	**Exemplary quotes**
**1.1) Consequences for the availability of care**	
1.1.1) Varying availability *	*In principle, there’s a gradient from north to south, the more southern, the more Catholic, the more difficult it is to find a possibility for an abortion. The [name of the city] is about mid-position [in Germany], it actually works quite well here. […] In the Rhineland [the care situation] is great. In the south of Germany [it's] catastrophic. The [patients] sometimes drive 150 kilometres to find someone to perform a termination.* Physician 7, Sections 24/ 89, prenatal diagnostician, inpatient sector, approximately 14 years of professional experience
1.1.2) Urban-rural divide *	*Interviewer: How do you perceive the current situation [in Germany] regarding the provision of abortions?* *– As pretty bad. And then, if at all, as almost sufficient in the cities. And in rural areas as rather catastrophic. Yes, definitely not comprehensive.* Physician 3, Section 178, ObGyn resident, 4 years of professional experience
1.1.3) Patients seeking care abroad *	*But it is of course a catastrophic situation that we are then forced to send women out of the country to Spain or England or France, depending on how far away they are, in order to have a foeticide at all.* Physician 23, Section 17, in- and outpatient sector, approximately 20 years of professional experience
1.1.4) Limitations in availability*	… due to the right to refuse* *It remains legally possible to opt for a later abortion, even up until the onset of labour, if there is a medical reason—specifically, if the diagnosis is incompatible with the current or future life situation of the pregnant woman. That is roughly how it is phrased in the legal text. The child itself is not considered a “harm” and does not appear as the justification for the abortion; rather, it is the woman’s life circumstances that are taken into account.* *At the same time, it is stated that “no one can be obliged to participate in an abortion”—whether it be the anaesthetist, the gynaecologist, or other medical staff. And this, I believe, poses a significant problem. Even those still in training—who witness these situations—are allowed to refuse to take part, and I don’t think this can be regulated. Those in medical training also have the right to opt out of participating in abortions.* Physician 22, Section 82, inpatient sector, approximately 25 years of professional experience **… due to the institutional use of the right to refuse**^**+**^ *And of course, this right to refuse—set out in §218a of the Pregnancy Conflict Act—is actually an individual right, meaning that a person can choose not to participate in an abortion. However, it is still being claimed and applied by entire institutions, hospitals, or senior consultants, effectively extending that right far beyond individuals. This even happens in academic teaching hospitals and non-religious facilities. So this arbitrary handling of the refusal right is also a major problem.* Physician 23, Section 77, in- and outpatient sector, approximately 20 years of professional experience
	… at higher gestational weeks* *But beyond three months [of pregnancy], it is practically impossible [to find a facility for an abortion] and then finding a solution for the women [...] becomes extremely difficult. And then there are always [...] at least two colleagues working intensively for several days to find a solution. And that's also so frustrating because we then have to represent the woman's concerns elsewhere. Whether it's with clinics, whether a psychiatric indication is needed or something like that and the others are so distanced. In other words, we call and say ‘We have a woman who is [...] 19 weeks pregnant.’ And then the practice says ‘Yes, but we won't have the next appointment for another three weeks.’* Physician 4, Section 56, outpatient sector, approximately 40 years of professional experience
	… in Dilatation & Evacuation* *[It is also important to me] that [the patients pregnant in] later weeks can ultimately decide whether [the pregnancy] is [terminated] surgically or with medication. After all, there are clinics that force patients to expulsion [of the pregnancy] due to a lack of expertise or interest. Some [patients] are made to expulse as early as the twelfth week. And then the women are told that it won't work, that there are no other options, right up to the prenatal diagnosticians in private practice. I recently had the same experience in a clinic in [another district]. I had a patient there who was 14+1 [weeks pregnant] or so, and [her physician] told her that it would be [...] a treatment error [...] to do an operation in that gestational week. She had no risk factors, so that was completely absurd.* Physician 23, Section 37, in- and outpatient sector, approximately 20 years of professional experience
	**… in palliative birthing options**^+^ *But it is not clear when you are supposed to perform foeticide [...]. Our [local] senior public prosecutor's office has interpreted that a stillborn child is absolutely necessary. When I read the law’ I'm not so sure about that. Of course, that's how we do it now [...]. But actually, I sometimes think that if [the child] dies in ’um's arms, it might not be so wrong. I think it's up to each clinic to decide for itself when to perform foeticide.* Physician 6, Section 99, inpatient sector, approximately 15 years of professional experience
**1.2) Consequences for the accessibility of care**	
1.2.1) Longer waiting periods^*^	*I find this care situation challenging [and] that it's obviously just extremely badly organised here in [this city] and the surrounding area, that patients simply have to wait a long time, that they won't get quick appointments, that they won't get quick diagnoses and then also won't get a quick appointment for a termination.* *I think it's a catastrophe for the patients, because [...] they are of course in the worst situation of their lives and simply have to stay in this situation for longer than is necessary.* Physician 10, Section 43, 37, inpatient sector, approximately 7 years of professional experience
1.2.2) Longer distances^*^	*So I was actually more moved by the fact that I've experienced several times that the care situation is very bad. Especially here in [this city] [...].* *We then asked all the other centres here whether it would be quicker and no one could give us an appointment any sooner. And there are only a few centres that still do that here in [this city]. [...] And yes, that was, that was a very heavy burden for my [relative], that she was actually left alone with it for such a long time. And then she actually drove to [city 450 kilometres away] because she got an appointment [there] the next day for the abortion. [...]* *I think it's also an issue [of] humane [treatment] to prioritise this [...] What we were told at the university hospital, yes, they could understand it. [...] And I wonder if that's really the case? I mean, this operation takes 15 minutes’ I'm a gynaecologist myself. I know how long something like this takes. Of course it takes a bit longer with all the [preparation] and so on, but in the end it's a quick operation.* Physician 10, Sections 8 ff. and 54, inpatient sector, approximately 7 years of professional experience

Exemplary quotes show causes and consequences of varying availability of abortion care as well as the right to refuse. The first column shows the sub-categories of the main category ‘availability’ and the second column corresponding exemplary quotes. Deductively derived categories are marked with an asterisk (*), and inductively derived categories with a plus (+)

**Table 2:** Main theme ‘legal regulation and stigmatisation’

**Categories ** **and sub-categories**	**Exemplary quotes**
**2.1)** **Consequences for the accessibility of care**	
**2.1.1)** Deficits in information transparency* …	… for patients* *[W]e do a lot of prenatal diagnostics in Germany and, of course, occasionally the pregnancy is found not to be healthy [...] and if the findings are very blatant, then the women are advised to have an abortion or they decide to have one. And then there is this gauntlet of ‘Who will do the abortion? Where can I go? Isn't it too late? Does an ethics committee have to decide?’* Counsellor 5, Section 12, medical physician, *Pro Familia*, approximately 30 years of work experience
	… due to language barriers* *Yes, often the linguistic aspect, so when women who won't have German as their mother tongue. Because then the subtleties of the language are often important. Especially when it comes to emotions. Because the women may be able to speak everyday German, but then telling them how they feel, what that triggers in them, that's often difficult.* Counsellor 1, Section 82, diaconal social services, approximately 30 years of professional experience
	**… for personnel** ^+^ *It’s the same with later-stage abortions—regional structures have developed around them as well, but no one really knows about them [including healthcare professionals] unless you’re directly involved or happen to be in the know.* Physician 23, Section 49, in- and outpatient sector, approximately 20 years of professional experience
	**… due to lack of professional networking** ^+^ *So it is usually the case that information is then passed on via the prenatal diagnostic practices. But even that is not guaranteed. It also varies from region to region. Or that certain clinics have established themselves in such a way that they mainly perform foeticides and others do not. And some standards have then developed internally. But these are non-transparent and inflexible.* Physician 23, Section 49, in- and outpatient sector, approximately 20 years of professional experience
** 2.1.2) ** Intransparency of medical decision processes due to third party involvement* …	**… concerning medical indication** ^+^ *But even there [in the clinic in the vicinity that perform foeticides], in [cases] where there is actually a medical indication in the mother's favour, [...] [the] prenatal diagnosis and [the] embryopathic indication [are] not sufficient in the assessment of these individuals, who have the power to carry out the foeticide or not. And then the women wander around and don’t get the foeticide, once they have been rejected, then they are also rejected elsewhere, because the others have already rejected it. Then [in addition] there's a loyalty skirmish.* Physician 23, Section 49, in- and outpatient sector, approximately 20 years of professional experience
	… concerning the involvement of ethics committees * *And then of course there are also ethics committees in many regions or in clinics where they are suddenly proud of the fact that they have such ethics committees, which is of course absurd because it completely ignores the legal situation. Then the woman can no longer describe her plight to the physician giving the indication, but instead there is a whole committee that decides whether the embryopathic situation fits the woman's suffering. So that's completely absurd. And if such ethics committees in some clinic have already said ‘No, we won't do it’, then all the other clinics to which the woman then turns say ‘No, we won't do it either, because they also said “no”’.* Physician 23, Section 49, in- and outpatient sector, approximately 20 years of professional experience
**2.2) ** **Consequences for the acceptability of care**	
2.3.1) Stigmatisation … *	… by personnel* *[One patient] had three children, she was in her early 40s and was expecting a child with trisomy and a heart defect. And the [counsellors] kind of pressured [her] with sayings like ‘Where there are three, there will be four’ and ... [thinking about it] well, in any case really, really pressured her to [carry the pregnancy to term] and made her feel very guilty, so that she was then quite desperate. So that was something I'll never forget because it was so dramatic. But I did have a few people from this counselling centre who said how much they were harassed.* Physician 23, Section 25, in – and outpatient sector, approximately 20 years of professional experience
	… by surroundings* *The second aspect is: once I have made my decision [as a patient], how do I communicate it? Do I stand by my choice, or do I acknowledge that the social pressure is incredibly strong?* *[…] But she herself was a paediatrician, coming from a background where abortions were not accepted.* *She ultimately decided to have the abortion with us at a late stage, in th* ^e^ *7th o* ^r^ *8th month. However, she simply communicated to her family and friends that it was sudden infant death syndrome—an intrauterine foetal death—and claimed she didn’t know what had caused it. So, it’s a difficult situation.* Physician 7, Section 32, prenatal diagnostician, inpatient sector, approximately 14 years of professional experience
	… due to internalised stigma* *It's not uncommon for women to say in prenatal care quite clearly [that they want an abortion]. A week later: [The] woman has had an induction of labour, is in labour, is in the delivery room and then says ‘I didn’t want this at all. I didn’t want this at all. They persuaded me to abort. I didn’t want this at all. I wanted to keep my child.’* Physician 7, Section 32, prenatal diagnostician, inpatient sector, approximately 14 years of professional experience
2.3.2) Psycho-emotional conflicts in patients/ partners *	*And even if it's not an extreme late-term abortion, but something like 16 or 18 weeks or something like that, the whole decision-making situation and the whole -’it's just a big, big drama and I haven’t actually seen a woman where it wasn’t a drama. Because they are simply in a huge conflict between ‘Can I trust in myself to have this child? How will I feel about it? Can I manage it?’ and the [termination of pregnancy].* Physician 18, Section 67, outpatient sector, approximately 28 years of work experience
** 2.3.3)** Lack of support by staff for patients decision* …	… for abortion* *The [patients get] prenatal diagnostics, and that very often and usually is carried out with a lack of information in such a way that it is carried out selectively. In the sense of a deficient perception of this developing life with the question ‘abort or not?’. So in the rarest of cases, people look at how to proceed therapeutically. And then there are many clinics that won’t help the women at all, where they then start an odyssey across the country and outside the country to see how they can get on, i.e. those who are already over 24 weeks and need a foeticide.* Physician 23, Section 17, in- and outpatient sector, approximately 20 years of professional experience
	**… for continuing the pregnancy** ^**+**^ *And I had a patient who came to me at the very end of her pregnancy and had abnormal findings in the prenatal diagnostics and didn’t want a termination under any circumstances and didn’t want any further diagnostics and of course actually wanted a healthy child. [She] was adamantly opposed to all those who wanted to carry out further diagnostics on her and the child ended up with Down’s syndrome. And she really, really had to fight her way through it to say ‘I want to carry this child to term as it is. And then I'll deal with the rest.’ [... She] was also prepared for the fact that she didn’t really need any support during the pregnancy. Because everyone blames her anyway for not having any further diagnostics and [not] terminating the pregnancy.* Physician 2, Section 74ff, inpatient sector, approximately 15 years of professional experience

The exemplary quotes show consequences of the legal regulation and stigmatisation of abortion in Germany. The first column shows the sub-categories of the main category ‘accessibility’ and the second column corresponding exemplary quotes. We chose quotes in accordance with best representing the category

Deductively derived categories are marked with an asterisk (*), and inductively derived categories with a plus (+)

**Table 3:** Main theme ‘lack of training and standardisation’

**Categories and sub-categories**	**Exemplary quotes**
**3.1) Consequences for the quality of care**	
3.1.1) Inadequate representation in residency *	… of abortion methods* *In* *terms of execution and implementation, there is a lack of many things. Firstly, there is a lack of training. There is a lack of training because it is not offered at all, it is a coincidence whether you get training or not, i.e. it is not offered systematically. If you are trained in Catholic centres, so to speak, you ’won’t learn that at all. I think that’s really bad, for example. ’you’re not trained at all, so you ’won’t systematically acquire the knowledge.* Physician 5, Section 46, outpatient sector, approximately 33 years of professional experience
	… of psychoemotional support* *I think we really need very well-trained, psychologically well-trained gynaecologists. I think many of them are already very good. And many are very good with women. I think what’s important, however, is that even the ones that do exist ’won’t fall into such a routine. I think that simply happens when you do a lot of abortions as a physician. Then it can become routine at some point. And of course there is also something of a routine in my profession. But nevertheless, the situation of women in pregnancy conflict is a situation in which it is very important to engage with the women again and again to a certain extent. Of course, this also requires a certain degree of mental hygiene on the part of the practitioners and the relevant professionals involved. But that would be desirable. So if I could make a few wishes from a fairy godmother, I would wish that for the physicians and also for the women.* Counsellor 19, Section 67, non-denominational provider, approximately 3 years of professional experience
	… due to limited training possibilities* *One of the main challenges is, first and foremost, getting into a position to learn in the first place, so to speak. And then, being able to practise in a safe and protected environment. After all, it is well known that this field of work, given its many opponents and controversies, can be particularly demanding.* Physician 3, Section 90, ObGyn resident, 4 years of professional experience
** 3.1.2) inadequate expertise in …**	**… Dilatation & Evacuation** ^+^ *So, really, from 22 to 24 weeks of pregnancy onwards, [D&E] is a standardised procedure in England, the Netherlands, the USA, and Spain. It is a well-established surgical method that, with proper training, can certainly be offered—there is no doubt about that. There are also evidence-based analyses supporting it.* *With Mifegyne and Cytotec, we now have optimal priming—cervical preparation has never been this safe in medical history. This allows for a controlled and gentle dilatation of the cervix, significantly reducing the risk of injury. Of course, foetal parts can still sometimes cause injuries, leading to a slightly increased risk. However, when compared to the fact that women have a choice—just as they do with all other medical procedures—this risk is acceptable.* *There are pros and cons, risks of injury, and various considerations, and these must be presented to the patient objectively. However, this requires the necessary expertise, which simply does not exist in Germany. That is a real problem: no one in Germany is properly trained in this. There must be adequate training to ensure that this methodologically sound equivalent is available.* Physician 23, Section 117, in- and outpatient sector, approximately 20 years of professional experience
	**… foeticide** ^+^ *Or we are back in the eminence of [anonymised], [leading] obstetrician at the [university hospital], who then says, for example, against the recommendation of the Royal College [of Obstetricians and Gynaecologists], that it would be [nicer] if the potassium chloride were only injected into the amniotic cavity, i.e. the amniotic fluid and not intracardially. And again, nobody says anything. On the one hand, it is also ethically questionable whether it is really so nice or only so nice for him to expose the women to the fact that they have to have an ultrasound every few hours and have to see whether [the foetus] is really dead. So is it ethical? Is it evidence-based? [...] And there are also [...] no German guidelines, [there] is also no discourse about it. And everyone does what they want. And we really won’t have any evidence, especially not the practitioners. * Physician 23, Section 53, in- and outpatient sector, approximately 20 years of professional experience
	**… use of abortion medication** ^+^ *Even the clinics that do this have simply used poor [prostaglandins without prior application of misoprostol], i.e. because nobody is interested in them, then they have ancient procedures and so on, where they then dose [the prostaglandins] far too low, constantly and always orally, so it's just really unfavourable. [...]* *Yes, but I think I already started working through it methodically during my specialist training in [anonymised] and found it terrible and horrible that women in Germany spend five days and I don't know how long in clinics, because they were only given prostaglandins and Cytotec and in some cases Nalador, so really, what had long been available at the time, where there had long been studies, very large-scale studies from Scotland, was completely ignored here, there was zero implementation.* Physician 23, Section 101, in – and outpatient sector, approximately 20 years of professional experience
3.1.3) Higher risks in complications*	*The only area where it becomes difficult is when the pregnancy size has progressed somewhat [...] and when you get to the edge of these possibilities, then of course it becomes challenging to realise this for the woman, on the one hand in terms of time, and on the other hand it is also associated with greater surgical effort: higher bleeding risk for the woman et cetera. * Physician 20, Section 28, inpatient sector, approximately 15 years of professional experience
** 3.1.4) Inadequate abortion related competencies required in other areas of obstetric care** ^+^	*And ultimately it also has a certain training effect, because you learn certain techniques for the [pregnancies] that are terminated in the foreseeable future, with which you can then carry through the healthy [pregnancies] that need to be saved, [for example] umbilical cord puncture. What we usually do in the department is that we don’t inject potassium directly into the heart, but into the umbilical cord vein. [...] And that is technically incredibly difficult. [...] If you have a child now and it has severe anaemia […] you can save it by giving the child a blood transfusion in the mother’s womb. [...] If you make a mistake, the child will die. This means that you will never be allowed to practise in such a situation for the first time. But if you have already performed 15 foeticides before, i.e. umbilical cord punctures to inject potassium, you will have a certain clinical routine and will be able to do it much more professionally without complications when you have the first foetuses to be saved, so to speak. And that’s always a bit of a justification or why people say ‘No, to be a good prenatal physician, you have to perform foeticides’. * Physician 7, Section 49, prenatal diagnostician, inpatient sector, approximately 14 years of professional experience *And it's also the case that if you decide to become a perinatal physician, you need different numbers of amniotic centeses and the easiest way to get them is of course to perform foeticide. But then, of course, you are also the one who actively performs the abortion. That’s what’s happening right now with a colleague who has decided to do prenatal medicine as a specialist training course and who is now easily getting the amniocenteses through foeticides. Yes, and others who say I want to become a perinatologist but don’t want to do foeticides, then struggle to get the numbers of amniocenteses.* Physician 8, Section 20, ObGyn resident, inpatient sector, 6 years of professional experience

Exemplary quotes show consequences of the lack of training and standardisation in abortion care in Germany. The first column shows the sub-categories of the main category ‘acceptability’ and the second column corresponding exemplary quotes. Deductively derived categories are marked with an asterisk (*), and inductively derived categories with a plus (+)

**Table 4:** Main theme ‘intersectional aspects’

**Categories and sub-categories**	**Exemplary quotes**
**4.2) ** Consequences for equity, inclusivity and patient-centredness of care *	
** 4.2.1) ** **multiple discrimination of** ^**+**^	**… patients with psychological conditions** ^+^ *When it comes to [...] high-week abortions for health, psychological reasons or whatever, it is extremely difficult to find physicians who will perform them. So medically it's still ok. But if it's done for psychological reasons, ’it's] extremely difficult. It was very different 10 years ago. And we are –– I'm [located] in a big city –– I'm not even talking about rural areas, otherwise they have to travel a long way to get it.* Counsellor 9, Section 86, non-denominational provider, approximately 28 years of professional experience
	… patients with histories of migration* *I want to prevent women from ending up in the hands of some bunglers at all cost, [...] people who feel authorised to carry out such a procedure on the kitchen table. [I] have experienced this myself . It's fatal, terrible for the woman, what happens there. Of course I want to prevent that.* *[...] this was a young, [non-German speaking] patient and she had presented for an abortion, somewhere in the [name of the city] area, but not at a physician. There was no medical documentation, no letters or anything like that. She had nothing, so to speak. And during this […] unprofessional abortion, pregnancy material was left behind, and the uterus was so badly damaged that the patient arrived in my care bleeding profusely and we then had no other option than to remove the uterus to save her life.* Physician 20, Section 40/ 53, inpatient sector, approximately 15 years of professional experience
	**… patients with risk of re-traumatisation** ^**+**^ *It was about a termination of pregnancy due to a medical indication in the 13th week. [...] And the waiting time at the university hospital was one week until the abortion. That would have been after the fourteenth week and [...] it would probably have ended for [her] in such a way that she would have had to give [still] birth to the child, which of course would have been an extremely heavy psychological burden for her. […]* *And that was a really, really heavy psychological burden, especially because [she] had been trying to get pregnant for four years and only got pregnant with several I[ntra] C[ytoplasmatic] S[perm] I[njection] attempts and then this diagnosis was so severe that the child might have died in the womb […].* *And having to endure this situation anyway is hard enough. And then to be left alone with it for such a long time, for almost a week. We finally went to the university hospital on Wednesday. They wanted to give us the appointment on Tuesday and the diagnosis had already been there since Monday. So that would have taken over a week.* Physician 10, Section 13, inpatient sector, approximately 7 years of professional experience *Yes, and of course where I always reach my limits is when we have these higher (weeks), which are not embryopathic, which then also need foeticide and nobody does it. [This was a woman] who fled from the [Middle East], raped several times, pregnant and very young, 22 [years old]] and 24 weeks [pregnant] and everyone rejects it and she's even in a psychiatric ward! That’s one of the most blatant cases! She was even hospitalised in a psychiatric ward and everyone rejected foeticide. All of them. It's so terrible. And you can talk and talk and talk to the heads of the clinics and yes: no chance. Sometimes I think I'll just do it myself, but then maybe that’s where fear and my other professional career come into play again. My own ambivalence is still holding me back, but that is certainly also inhibited by the political situation, yes, and stigmatisation.* Physician 23, Section 93, in – and outpatient sector, approximately 20 years of professional experience, highlight according to the interviewee’s emphasis
4.2.2) lack of patient-centredness*	… due to infringement of dignity* *In our practice, we do these abortions and* *then we send the women to the clinic we work with. And we also have a consultation hour every day at this clinic. And so I once asked my boss why it's not actually possible [...] to order the women directly to the clinic and then have the abortion there [...], so that they don't have to drive all the way through [city name] to go to the hospital to give birth. And my boss also said quite specifically ‘The hospital doesn't want that. Then it will end up in the press that abortions are being performed there.’ [...] it's more likely to be handled by the management or by everyone in general in such a way that it happens as quietly as possible and that the woman then simply comes there with a dead child practically in her womb and can then give birth. But there is no sensible reason why we now perform these abortions in the practice and then send the women to the clinic.* *[…] But [in my opinion] it's a moral issue, [that] people have [with abortion] and therefore the clinic is afraid that if it somehow becomes very public that they do a lot of abortions, then it could be that they somehow have a negative image in the city. So I think it's kind of sad that it's like that.* Physician 10, Section 94, inpatient sector, approximately 7 years of professional experience *To be honest, I [would like to have] rooms that do justice to this. We turn a double room into a single room, so to speak, and offer to let the partner stay. Of course, the health insurance companies refuse to pay for this. This means that eight months after the termination, they still get a bill at home. I find that terrible.* Physician 6, Section 117, inpatient sector, approximately 15 years of professional experience
	… due to infringement of bodily autonomy* *It was about a termination of pregnancy due to a medical indication in the 13th week. [...] And the waiting time at the university hospital was one week until the abortion. That would have been after the fourteenth week and [...] it would probably have ended for [her] in such a way that she would have had to give birth to the child, which of course would have been an extremely heavy psychological burden for her.* Physician 10, Section 13, inpatient sector, approximately 7 years of professional experience
** 4.3) Ethical consequences** ^+^	*Some [patients] even come from 200, 300 kilometres [away], they drive past two clinics that perform abortions, i.e. with foeticide. That's always the [problem], because the others didn't have any appointments beforehand and we were able to provide the quickest appointment in case of doubt. It's the same the other way round when in doubt. For our personal mental hygiene, we once said that we wouldn't do more than two foeticides a week and then some of them would wait two or three weeks for the procedure. This is of course psychologically horrible and they look for other clinics, some of which are far away.* Physician 6, Section 89, inpatient sector, approximately 15 years of professional experience

Exemplary quotes show intersectional consequences of aforementioned themes on abortion care in Germany. The first column shows the sub-categories of the main category ‘equity, inclusivity and patient-centredness of care’ and the second column corresponding exemplary quotes. We chose quotes in accordance with best representing the category

Deductively derived categories are marked with an asterisk (*), and inductively derived categories with a plus (+)

### Varying availability and the right to refuse

Experts described abortion availability in Germany as varying (Eq. 1.1.1, Table 1) and highly dependent on the number of abortion providers, noting a decline in abortion services over the last decades:

*But we are already noticing that the physicians who have been performing abortions for a long time are simply getting old and then giving up their practices*,* retiring and younger colleagues [are] taking over the practices but not performing abortions for a variety of reasons. In other words*,* there is simply a stealthy extinction of abortion care.*

Counsellor 6, Sect. 12, Pro Familia, 10 years of professional experience.

Experts observed a divide between urban and rural regions (Eq. 1.1.2) and increasing numbers in patients seeking care abroad (Eq. 1.1.3). The availability and accessibility of medically indicated abortions was rated as inadequate to poor, in some cases even *“*catastrophic*”* (Eq. 1.1.1–1.1.3). The experts highlighted a severe shortage of facilities offering abortion beyond 14 weeks’ gestation (Eq. 1.1.2), D&E or palliative birthing options, therefore limiting choices in method for patients. These limitations in availability in turn result in access barriers, such as longer waiting periods and distances for patients (Eq. 1.2.1–1.2.2).

The generally low number in abortion providers in Germany was attributed to the overall criminalisation whilst the decline was attributed to a persistent stigmatisation of abortion (see Sect. "Legal regulation and stigmatisation" ).

Another reason cited by the experts was the common usage of the right to refuse. Some experts acknowledged this right to refuse as protecting physicians from being forced into procedures that conflict with their personal beliefs. They saw it as a legal instrument to protect a diversity of perspectives on abortion. At the same time, they highlighted its consequences, arguing that if many providers opt out, this exacerbates provider shortages and workload for remaining providers, resulting in prolonged waiting times and delayed procedures, therefore increasing emotional and physical burdens for patients.

Moreover, they described the phenomenon of ‘institutional refusals’: They explained that entire departments – i.e. obstetric and anaesthesiologic departments – as well as entire hospitals declined participation in abortion care, sometimes even regardless of indication – including Christian as well as publicly funded institutions, University and teaching hospitals:

*We have two district hospitals nearby. One of them is quite progressive in terms of abortion care: the head of gynaecology would be willing to perform abortions. But the head of anaesthesiology categorically refuses to take part. The fact that this is possible in a district hospital that is publicly funded—I find that unacceptable. And I see that as one of the future challenges: that this simply cannot be allowed to happen. […]*

*And it goes so far that I had to refer a patient [pregnant] with [a foetus diagnosed with a lethal condition]—who had already given birth to two children in that hospital*, *and who would have liked to have her abortion there because she felt very comfortable there—to a different facility.*

Physician 21, Sect. 48 ff, outpatient sector, approximately 30 years of professional experience.

The experts interviewed evaluated this as further restricting availability and accessibility of (medically indicated) abortions. Therefore, they were very critical of institutional refusals.

### Legal regulation and stigmatisation

Interviewees linked the decline in abortion providers to demographic changes, legal regulations and persisting abortion stigma. While medically indicated abortions are formally legal, their inclusion in the Criminal Code reinforces a sense of criminalisation. Experts also noted that gynaecologists performing abortions often feel legally insecure:

*But the exact point in time when a foeticide should or should not be carried out is not clearly defined in legal terms. Our public prosecutor’s office has interpreted the law to mean that a stillborn child is absolutely necessary. But when I read the legislation myself*,*I’m not so sure about that. Of course*,*we now comply—we ensure that the child is born dead. But sometimes I wonder whether it wouldn’t be just as acceptable if the baby were to pass away in the mother’s arms.*

*This timing—when exactly a foeticide is performed—I believe every hospital has more or less decided that for itself. And then there are those 500 instances of “no penalty if…”—and you find yourself thinking*,*“Well*,*I hope I’ve filled out the very last form correctly and haven’t forgotten anything.” Sometimes those forms are filled in just to have them done*,*not necessarily because they reflect something of meaningful substance.*

Physician 6, Sect. 101, inpatient sector, approximately 15 years of professional experience.

Moreover, experts described changes in attitudes towards abortion amongst different generations of gynaecologists: They described a shift of an older generation socialised in the 1970 s during the Women’s movement towards a more conservative middle-aged generation. The latter was described as more restrictive in acceptance of abortion and often associated with stronger stigmatising behaviour towards patients and colleagues. This generation constitutes the personnel currently occupying key positions in the healthcare system and decision-making regarding educational content, while the older generation is increasingly retiring. A younger generation of (aspiring) medical professionals is described as more open to the topic and is positively recognised for its destigmatising educational efforts. However, it might not be able to fill the staffing gaps in light of demographic change, according to the interviewees.

Apart from influencing the personnel’s willingness to offer abortions, the legal regulation and stigmatisation of abortion also affected its accessibility: According to the interviewees, there are deficits in information transparency both for patients (Eq. 2.1.1) as well as personnel. One reason experts attributed this intransparency to were language barriers for patients. Another one were legal restrictions, particularly § 219a of the Criminal Code, which previously limited public information on and by abortion providers. Another reason cited was a lack of networking amongst healthcare professionals, when it comes to referring patients from prenatal diagnostics to abortion providers if needed.

Moreover, interviewees described a lack of transparency when it comes to medical decisions processes, mainly due to third party involvement (Eq. 2.1.2). Some interviewees strongly criticised the involvement of ethics committees in this context, arguing that once a committee denied a patient an abortion, finding an alternative provider became significantly harder.

A key theme in the interviews was the persistent stigmatisation of abortion in both society and the medical system (Eq. 2.3.1). This manifests in patient stigmatisation by healthcare personnel (including physicians and midwives), by personal surroundings, and patients’ own internalised stigma.

Internalised stigma, according to the interviewees, also stems from conflicting societal expectations regarding parenthood, decisions around pregnancy and a child’s potential disability. According to them, patients experience a discrepancy between the social expectation to give birth to a child considered ‘healthy’ by societal standards, and society’s structural ableism. These contradictions that are often reflected in the attitudes and remarks of medical professionals contribute to emotional conflicts and psychological distress (Eq. 2.3.2). The experts described these inconsistencies as a double standard: the society and some physicians expect pregnant persons to undergo prenatal diagnostics, yet some facilities offering these tests refuse to provide abortion care if termination is chosen. Interviewees described that sometimes this leads to a lack of support by professionals for patients in accessing abortion care (Eq. 2.3.3). Conversely, sometimes patients who decline prenatal diagnostics or decide to continue their pregnancy despite a potential foetal diagnosis encounter limited support for continuing the pregnancy.

### Lack of training and standardisation

Interviewees stated that the current legal regulation negatively impacts the quality of abortion care and limits its representation in medical education and obstetric training (Eq. 3.1.1, Table 3) –– in consequence affecting both procedural and communicative skills to offer psychological and emotional support for patients and partners:

*[Abortion was] underrepresented [in medical school]. [I] can say that exactly*,* there were some two slides: this is done*,* what is the paragraph called? What do you have to do? Sure*,* counselling regulations. The usual. But nothing else in terms of the whole social situation*,* the whole background. How do I support the women emotionally?*

Physician 14, Sect. 62, inpatient sector, approximately 12 years of professional experience.

A major challenge reported by participants was balancing the right to refuse with the need to integrate abortion care into medical education. The experts questioned how proficiency in abortion techniques could be ensured when general abortion provider numbers are low, institutional objections to perform abortions are common and opportunities to learn abortion methods are therefore limited. They emphasised the role of training opportunities not only in skill development but also in fostering informed personal stances.

They reported that insufficient training leaves providers unfamiliar with internationally recognised abortion methods, raising concerns about the adherence to medical standards in D&E, foeticide and medication abortion (Eq. 3.1.2). This in turn results in a higher risk in complications in their point of view (Eq. 3.1.3).

Experts also attributed the lack in expertise to a lack of national guidelines on abortion^2^, particularly beyond 14 weeks’ gestation, but also a lack of sensitivity for the importance of abortion skills for other obstetric contexts. For instance, they highlighted that abortion procedures provide essential training for therapeutic interventions like intra-uterine punctures. Since these are first practised in abortion settings to avoid harming a viable foetus, physicians who refuse to provide abortions may take longer to qualify as prenatal diagnosticians. Experts therefore argued that the reduced number of experienced abortionists impacts prenatal care beyond abortion services.

The lack of standardisation also creates uncertainty in determining medical indications, as no clear guidelines define “sufficient” criteria. Without official standards for medically indicated abortions, providers set their own criteria, resulting in inconsistencies between facilities further adding to the intransparency of medical decision-making processes. Experts cited cases where people sought abortion due to prenatal foetal diagnosis, but providers deemed the condition “insufficient” to justify termination. They saw this as a shift of power from the patient to the providers that undermined the legal regulation.

The interviewees further noted that psychological and emotional support for patients is inconsistent, with referrals to counselling services depending on the initiative of individual facilities.

### Intersectional aspects

According to the interviewees, the intersection of the right to refuse, criminalisation, stigma, and the underrepresentation of abortion in medical training create systemic barriers to equitable, inclusive, and patient-centred care. The experts identified significant gaps in Germany’s healthcare system, with intersectional^3^[53] access barriers disproportionately affecting structurally disadvantaged groups: patients living in rural areas, caregivers, those facing language barriers, and individuals with migration histories (Eq. 4.2.1, Table 4) or pre-existing conditions such as mental health conditions. Moreover, interviewees warned of the risk of re-traumatisation for patients with past experiences of sexual trauma, child loss or fertility treatments as the following examples illustrate.

One interviewee highlighted the intersections of limitations in accessibility and lack of trauma sensitivity amongst personnel invoking the experiences of a relative: She needed a medically indicated abortion due to a fatal foetal diagnosis, but feared re-traumatisation in case of a stillbirth as she had experienced multiple miscarriages following in vitro-fertilisation. She therefore preferred surgical treatment but was nearing the end of the first trimester. As the availability of surgical abortion beyond 14 weeks’ gestation is scarce in Germany, time pressure increased. Despite being a trained gynaecologist and abortion provider as well as living in an urban area, the interviewee was unable to help their relative access a timely aspiration abortion nearby. They criticised that no provider they contacted acknowledged the urgence of a timely treatment.

Another interviewee cited an example to illustrate how opaque regulations on medical indications create inequities in care: They described an experience where a refugee, pregnant due to sexual violence, sought an abortion after arriving in Germany past 14 weeks. Despite her traumatic history and ongoing psychiatric care, this patient did not receive a medical indication and was unable to access abortion care. In this context, the experts stressed that, in practice, medical indications are often evaluated based on a foetal diagnosis, as in the former embryopathic indication, rather than based on aspects related to the pregnant person’s health, which might contradict the legal framework.

According to the experts, the right to refuse and insufficient abortion training limited overall availability of abortion care and choice of method in such a way that it in consequence undermined patient-centred care. One gynaecologist reported that while a nearby hospital refused to perform abortions, it accepted patients for stillbirth deliveries, forcing those seeking abortion to undergo foeticide at another practice before being referred to said hospital for labour. They evaluated this as undermining dignity in patient care and infringing on the bodily autonomy of patients (Eq. 4.2.2.).

Lastly, interviewees linked abortion care to broader systemic pressures in healthcare, exacerbated by the shrinking number of providers. This increases workload on remaining facilities, particularly for abortions beyond 14 weeks, leaving little time for patients’ and professionals’ psycho-emotional needs. The interviewees stressed the importance of psychological support for healthcare providers, particularly midwives, who often spend more time supporting patients through stillbirths or palliative births than physicians do. Experts also pointed out the ethical implications of the care situation (Eq. 4.3), in which high workloads emotionally strain the personnel and lead to treatment delays for patients due to access barriers, increasing risk of complications and limiting their choices of method.

## Discussion

This qualitative interview study provides insights into the perspectives of gynaecologists and abortion counsellors on the structural challenges of medically indicated abortion in Germany. The findings suggest that its regulation under the Criminal Code creates systemic barriers despite formal legality, reinforced by societal and professional stigma. These barriers affect all WHO criteria for abortion care and disproportionately impact marginalised groups. Additionally, criminalisation and healthcare working conditions hinder patient-centred care. In the following, we discuss our findings focussing on disparities in abortion availability and access, regulatory intransparency, the right to refuse, deficits in medical education and training, stigma, and multiple marginalisation.

### Disparities in abortion availability and access

Existing studies have highlighted regional disparities in abortion access in Germany, particularly due to the declining number of trained providers [25, 54]. This research focusing on first trimester abortions after mandatory counselling indicates an urban-rural divide. This urban-rural divide in abortion access is an internationally reoccurring phenomenon and has been described before, e.g. for Ireland [55], Australia [56, 57] or the US [58]. However, there is a lack of data specifically addressing access to abortions beyond 14 gestational weeks in Germany [35]. According to the German Federal Statistical Office [24], the distribution of patients undergoing abortions with foeticide varies markedly across federal states.

Our study confirms these disparities and adds qualitative nuance: while some participants described sufficient access in metropolitan areas, others—despite being healthcare professionals themselves—reported difficulties in obtaining appointments even in urban regions. Access becomes increasingly restricted as gestation progresses, especially for abortions for psychological reasons. This aligns with earlier findings suggesting a de facto privileging of indications based on prenatal foetal diagnoses over health concerns for the pregnant person [35].

Another barrier identified in our study is the disconnect between prenatal diagnostics and abortion care. Patients already embedded in the healthcare system due to foetal diagnoses often encounter significant delays or refusals when seeking abortion services. This fragmentation undermines care continuity and raises ethical concerns.

### Right to refuse

Our study demonstrates that the right to refuse has considerable impact on the provision of medically indicated abortions in Germany. The invocation of this right, especially for abortions beyond 14 weeks’ gestation, leads to a reduction in available services. International research into conscientious objection highlights negative impacts this right can have on healthcare systems and reproductive rights [59], such as delays in abortion access, particularly in rural areas [55, 59]. Inadequate regulation of conscientious objection can also increase emotional, psychological and physical strain on patients, reinforcing unequal access to care [59].

Our study also identified the institutional use of the right to refuse as a structural form of objection: hospitals – with and without denominational affiliation – refuse to provide abortion altogether. According to Merner et al. [60], this practice is poorly regulated and infringes on the rights of both patients and healthcare professionals, particularly when carried in publicly funded institutions – a criticism shared by the experts interviewed in our study.

### Regulatory intransparency on medical indications

Prior research [61] has shown that the legal framework for medically indicated abortions in Germany is characterised by ambiguity and inconsistency. Patients and providers alike struggle to interpret the criteria for medical indication, particularly when prenatal foetal diagnoses are involved. As a consequence, access often depends on informal negotiations and individual interpretation of legal thresholds [61], as supported by our results. Experts in our study reported considerable uncertainty in the practical application of the law. Moreover, they criticised ethics committees, intended to support complex cases, for gatekeeping abortion access, which can delay care and reinforce power imbalances between providers and patients. Both the *German Society for Gynaecology and Obstetrics* [62] and the WHO [29] advise against third-party authorisation, arguing it risks unnecessary delays.

Furthermore, our findings closely align with the results by Fields et al. who investigated abortion regulations and resulting ethical dilemmas faced by obstetrician-gynaecologists in Ohio [63]. They show how restrictive legal frameworks coupled with inconsistent institutional interpretations can lead to delay, ethical distress among providers, and compromise patient care. Physicians in Fields’ study reported feeling torn between professional obligations and fear of legal repercussions, mirroring the uncertainty described by German providers in our study. In both contexts, vague laws and institutional gatekeeping mechanisms contribute to fragmented care and jeopardise the timely provision of medically indicated abortions.

Provided they do not delay or restrict access, clinical ethics consultations could play a supportive role in reducing moral distress among abortion providers. Further research is needed to understand how such structures operate in practice and whether they might inadvertently impact access to care.

### Deficiencies in medical education and training

Several studies have identified gaps in abortion-related medical education and training in Germany [35, 45, 64]. There seems to be an imbalance in the availability and use of different abortion methods, with abortions after 14 weeks of gestation overwhelmingly performed medically [24]. Moreover, German health statistics lack detail on surgical abortion methods at higher gestational age and foeticide procedures, limiting the evaluation of care quality [24]. Aligning with this, our results indicate that training opportunities are sparse, particularly for D&E and foeticide. This might restrict the number of competent providers and centralise services in a few urban centres, exacerbating access disparities. Similar training gaps both in technical and psycho-emotional aspects of abortion care have been described for other countries, e.g. Ireland [65, 66]. Potential impacts of these deficits on pregnancy-associated risk of mortality in Germany cannot be comprehensibly assessed due to the lack of data on gynaecologists’ abortion skill sets and the insufficiently detailed and systematic recording of pregnancy-associated risk of mortality [67, 68].

### Stigma

International research has documented the multifaceted stigma surrounding abortion, affecting both patients and providers [42, 44]. Experts in our study highlighted how stigma operates across social, professional, and institutional contexts, therefore confirming Millar’s thesis [43] from a provider’s point of view that abortion stigma should be understood as a structural problem rather than an individual one. Interviewees partly reproduced abortion stigma by framing it as an exceptional procedure, therefore also confirming the multidirectional dynamics of abortion stigma as described by Millar [43, 69].

Moreover, the experts interviewed in our study pointed to the intersection of abortion stigma and ableism, particularly criticising ableism within the healthcare system. Different studies support that societal ableism and negative attitudes towards disability influence clinical decision-making and institutional policies in Germany [61, 70]. Importantly, our findings underscore how stigma impacts those seeking abortions for mental health reasons or in the context of prenatal foetal diagnoses. From a medical ethics perspective, the tension between the routine use of medical indications for abortion in cases of a foetal diagnosis and the pervasive influence of societal ableism is particularly troubling. Therefore, we support the expert commission’s suggestion to reform the legal framework to increase transparency in abortion regulations [18].

### Multiple discrimination of marginalised groups

International evidence suggests that marginalised groups — particularly people with mental health conditions, histories of trauma and/or migration — face greater barriers in accessing abortion and perinatal healthcare [3, 4, 71, 72] (also see [73]). While quantitative data for Germany is scarce [35, 74], our findings indicate that these groups also face disproportionate barriers to abortion care in Germany as well. Experts described how intersecting forms of discrimination compound existing access issues based on mental illness, language barriers, or precarious legal status. Participants criticised the lack of structural support for vulnerable patients, and the failure to provide inclusive and trauma-sensitive care. The systemic barriers identified undermine not only the accessibility of care, but also threaten the ethical principles of the respect for patient autonomy and justice. While our study indicates that individuals with a personal history of migration are particularly disadvantaged by the current legal framework, there is no structural response to this issue in Germany. At present, this gap is primarily being addressed by civil society organisations such as *Women Help Women* [75] and *Ciocia Basia* [76], which support people from countries with restrictive abortion laws and/or with migration histories in accessing abortion care in Germany.

### Strengths and limitations of the study

When interpreting the study results, several limitations should be considered. First, despite our efforts to phrase the call for participation neutrally, experts with particular awareness of abortion care challenges may have been more inclined to participate. Also, even though physicians were explicitly recruited independent of whether they themselves provided abortion care, only physicians who either performed abortions or provided counselling took part in the study. Despite extensive efforts, we were unable to include general practitioners/family physicians. Their insights would be valuable, particularly regarding long-term patient needs and challenges.

Our findings are shaped by the positionality and disciplinary backgrounds of the research team. While the study maintained a reflexive approach throughout its design, data collection and analysis, we acknowledge that our interpretative lens may have influenced the emphasis placed on certain themes, particularly those concerning institutional norms and ethical considerations. Given our personal stance on abortion as an essential part of sexual and reproductive health care, there is a potential interpretative bias in the analysis of the data. We sought to mitigate this through iterative coding, peer debriefing, but recognise that alternative interpretations are possible.

Moreover, participants were recruited regardless of their stance on abortion and whether they worked in secular, denominational, or public institutions. This enabled the inclusion of a variety of viewpoints, including critical perspectives on abortion. Importantly, it is notable that the description of challenges did not significantly differ between differing personal stances on abortion.

In spite of the described limitations, this study gathered an extensive sample, with 42 experts from diverse professional backgrounds and geographical locations. This allowed for the representation of varied perspectives across different regions in Germany, taking into account interregional legal differences. The study included experts from inpatient and outpatient settings, as well as both abortion providers and counsellors, capturing a broad range of insights.

The combined inductive-deductive approach to the analysis yielded valuable insights that went beyond the initial research questions. While the study initially was framed around the state of and challenges in abortion care in Germany, the inductive coding process revealed deeper structural dimensions shaping the German abortion care landscape. Notably, interviewees frequently described an implicit institutional culture of avoidance surrounding abortion provision, such as reputational concerns, internal “no-abortion” policies, informal disincentives for staff participation – issues that would likely have been missed in a purely deductive analysis. Furthermore, the inductive approach brought attention to the disconnect between prenatal diagnostics and abortion services, raising questions about systemic fragmentation that had not been central to the original research design.

## Conclusions

Our results suggest that medically indicated abortion care in Germany is constrained by legal ambiguity, fragmented service provision, and the common use of institutional refusal.

Institutional and interpersonal stigma remains a key barrier to abortion care, reinforced by its regulation under the Criminal Code, insufficient professional training, and generational differences among providers. Marginalised groups, such as pregnant persons in rural areas, migrants, and those with histories of trauma, face particularly high obstacles.

Our study highlights how regulating abortion under the Criminal Code creates barriers for both patients and providers. Although the legal provision in Germany aims to protect against disability-based discrimination, it has unintended consequences for those facing complex reproductive decisions. Our findings therefore support the expert commission’s call to reassess the definition of medical indication. Additionally, while the right to refuse protects providers, its opaque use can exacerbate inequalities and delay access to care. Thus, the lack of regulatory clarity around the right to refuse—especially at the institutional level—warrants closer examination.

Future legal frameworks need to protect against discrimination at all stages of pregnancy and ensure equal access to abortion care, regardless of social status, health condition, migration history, or language background. Additionally, national clinical guidelines on second and third trimester abortion care are urgently needed. Systemic reform, legal clarity, and improved medical training are essential to ensure equitable, patient-centred abortion care in Germany.

## Supplementary Information

Supplementary Material 1.

Supplementary Material 2.

## Data Availability

To protect the experts’ identities, the full interview transcripts are not publicly available. Supporting quotations can be found in Tables 1, 2, 3 and 4 within the manuscript.

## Abbreviations

UN: United Nations
WHO: World Health Organisation
D&E: Dilatation and Evacuation

## Footnotes

1 The data does not reflect precise provider numbers or available methods: Institutions in Germany that perform abortions are legally obliged to report the procedures performed for statistical purposes. These reporting institutions are documented by the Federal Statistical Office, but not the number of individual providers performing abortions at each institution [34, 35].

2 The first German medical guideline on first-trimester abortion was published in 2022 [47], after the interviews for this study had already been conducted. It focuses on abortions following the counselling regulation. There is currently no German guideline addressing abortions beyond the first trimester and/or those performed for medical reasons

3 We refer to intersectionality as defined by Kimberlé Crenshaw [53] for understanding how multiple forms of discrimination overlap and interact, particularly in the lives of marginalised individuals. Crenshaw coined the concept to describe how Black women face both racial and gender discrimination simultaneously, which cannot be understood adequately by looking at racial or gender discrimination alone.
