# Supplementary material for: Structural barriers to medically indicated abortion in Germany: A qualitative study of provider perspectives
Source: Reprod Health. 2025 Dec 10;22:253. doi: 10.1186/s12978-025-02116-9 (PMC12709803; doi:10.1186/s12978-025-02116-9)
Supplement: Supplementary file 1 — Supplementary Material 1. [file 12978_2025_2116_MOESM1_ESM.pdf]

Structural barriers to medically indicated abortion in Germany: A qualitative study of provider perspectives

Amelie Kolandt[1]*, Susanne Michl[1], Mirjam Faissner[1]

[1]Institute of the History of Medicine and Ethics in Medicine, Charité - University Medicine Berlin, Berlin, Germany

***Corresponding author**: amelie.kolandt@charite.de

Appendix 1: Materials and Methods in accordance with Consolidated criteria for reporting qualitative research

Research Team and Reflexivity

Personal Characteristics

The interviews were conducted by Amelie Kolandt (AK), who has a background in musicology and medicine. She completed both basic and advanced training in qualitative research at *Charité* and gained initial research experience during her undergraduate studies in musicology. From 2017 until 2021, she was a volunteer member of *Medical Students for Choice Berlin*. Throughout the study, she was supervised by Susanne Michl (SM) and Mirjam Faissner (MF), with backgrounds in history, medical humanities, medicine, and philosophy. Both have extensive experience in qualitative research. Regular participation in research colloquia at *Charité* and *Medizinische Hochschule Brandenburg* provided additional support for analysis and interpretation.

Relationship with Participants

Participants were initially contacted via email, where they received detailed information about the study. Before the interview, the interviewer introduced herself and explained her educational background via telephone. Participants were informed about the research project and the interviewer's training in qualitative methods. AK's involvement in advocacy groups related to reproductive rights was critically reflected upon in discussions with supervisors and in research colloquia to ensure research neutrality.

Study Design

Theoretical Framework

The study employed qualitative content analysis following Mayring's approach, using a combination of inductive and deductive methods. This method was chosen for its systematic

and transparent approach to analysing extensive interview data. Thematic structuring was guided by United Nations and World Health Organisation (WHO) quality criteria for healthcare provision.

Participant Selection

A nationwide recruitment strategy was used. Experts in gynaecology, obstetrics, and pregnancy conflict counselling were invited to participate. Written invitations were sent to professional organisations, medical societies, counselling services, and public health institutions. Recruitment advertisements were also placed in professional journals, and a snowball sampling method was applied.

Method of Approach

Participants were initially contacted via email and provided with detailed study information, including objectives, procedures, and data protection measures. Telephone inquiries were accommodated if needed.

Sample Size

A total of 42 experts participated:

- 20 specialists in gynaecology and obstetrics
- 2 doctors in advanced training for gynaecology and obstetrics
- 20 state-certified pregnancy conflict counsellors

Non-Participation

Five initially interested experts did not participate due to ineligibility, scheduling conflicts, or non-responsiveness.

Setting

Setting of Data Collection

Interviews were conducted via telephone. The interviewer ensured an undisturbed environment, while participants chose their preferred setting. Some were alone in their offices, while others participated from clinical contexts, occasionally experiencing minor interruptions.

Presence of Non-Participants

No other individuals were present during the interviews, although some participants had colleagues in the background.

Sample Description

Participants included medical specialists and state-certified counsellors. They worked in various healthcare settings, including outpatient and inpatient services. The sample was

diverse in terms of professional experience, institutional affiliation, and geographic distribution across Germany.

Data Collection

Interview Guide

The interview guide was developed based on Helfferich's [3] methodology. It covered topics such as professional background, daily work routines, challenges in abortion care, and future perspectives. It was pilot-tested and adjusted accordingly. A sociodemographic questionnaire collected additional background information.

Repeat Interviews

Participants were offered the opportunity to provide supplementary interviews for clarification or additional input, but none opted for this.

Audio and Video Recording

All interviews were digitally recorded and transcribed verbatim using *Happyscribe*. Transcriptions were manually corrected and pseudonymised by the lead author.

Field Notes

Field notes were taken during the interviews to facilitate follow-up questions and deeper exploration of participants' statements.

Duration

Interviews lasted between 25 and 105 minutes, with an average duration of 50 minutes.

Saturation

Data collection followed the principle of theoretical saturation. No new themes emerged in the final stages of coding, indicating that thematic saturation had been reached.

Transcripts Returned

Participants were given the opportunity to review and correct their transcripts, but none chose to do so.

Analysis and Results

Data Analysis

The qualitative content analysis process followed Mayring's approach [40] involved multiple readings of the transcripts, paraphrasing, and the development of thematic subcategories. Coding was initially conducted by AK, with 25% of the material counter-coded by two additional researchers. Interpretive uncertainties were discussed in research colloquia, and the coding

system was refined accordingly. The final coding framework aligned with WHO healthcare criteria: availability, accessibility, acceptability, quality, and equity/inclusivity/patient-centredness.

Description of the Coding Tree

A structured coding framework was developed, with categories and definitions outlined in Table 1–5.

Derivation of Themes

Themes were derived through a combination of inductive (emerging from the data) and deductive (aligned with WHO healthcare criteria) methods.

Software

Data was analysed using *MaxQDA* 2022.

Participant Checking

No additional feedback from participants was sought beyond the opportunity to review transcripts.

Quotations Presented

Selected participant quotations are included in the results section.

Data and Findings Consistency

The findings were systematically analysed to ensure consistency with the data
