# Supplementary material for: Structural barriers to medically indicated abortion in Germany: A qualitative study of provider perspectives
Source: Reprod Health. 2025 Dec 10;22:253. doi: 10.1186/s12978-025-02116-9 (PMC12709803; doi:10.1186/s12978-025-02116-9)
Supplement: Supplementary file 2 — Supplementary Material 2. [file 12978_2025_2116_MOESM2_ESM.pdf]

# Structural barriers to medically indicated abortion in Germany: A qualitative study of provider perspectives

Amelie Kolandt[1]*, Susanne Michl[1], Mirjam Faissner[1]
[1]Institute of the History of Medicine and Ethics in Medicine, Charité - University Medicine Berlin, Berlin, Germany
***Corresponding author**: amelie.kolandt@charite.de

## Appendix 2: Interview Guide and Sociodemographic Questionnaire

### Interview Guide

1. What motivated you to participate in this conversation today?
2. What role does the topic of abortion play in your daily work?
   2.1. Is there a standard procedure when someone comes to you who is unintentionally pregnant? (Can you describe it?) *
   2.2. How often do you conduct abortion consultations or abortion counselling? *
3. What challenges do you see in connection with abortions?
   3.1. Do you have experiences or ideas on how to address these challenges? *
4. Have you ever faced conflicts—internal or external—regarding abortions?
5. Do you get the impression that abortion is seen as a routine procedure in your field of work?
6. Can you recall whether and how abortions were addressed in your education or training?
7. How would you assess the current situation regarding abortion care?
   7.1. And in Germany/in your region? *
   7.2. What do you think the future of abortion care will look like? *
   7.3. In your opinion, should this development be addressed? *
8. What are your wishes and/or visions for the future regarding this topic?
9. What are your fears or concerns about this topic?
10. Is there anything you haven't had the chance to mention yet but would like to add?

Questions marked with * were only asked if they weren't already answered during the course of the conversation. The order of the questions could vary depending on the flow of the interview.

## Sociodemographic Questionnaire

1. Age (open field)
2. Gender: male, female, other, no disclosure (options to check)
3. Details about education and training:

   3.1. When and where did you study?

   3.1.1. Study location(s) (open field)

   3.1.2. Years from (open field) to (open field)

   3.2. When and where did you complete your (specialist) training, if applicable?

   3.2.1. Training location(s) (open field)

   3.2.2. Years from (open field) to (open field)
4. Details about your work routine:

   4.1. How would you characterize your work environment? Urban/rural (options to check)

   4.2. How long have you been working as a doctor/counsellor? Indicate in years (open field)

Multiple responses were not possible.
